# Distinct Plasma LPC Signatures Differentiate COVID-19 Sepsis from Other Sepsis Aetiologies

**DOI:** 10.3390/biomedicines13092110

**Published:** 2025-08-29

**Authors:** Vlad Pavel, Patricia Mester, Marcus Höring, Gerhard Liebisch, Stephan Schmid, Martina Müller, Christa Buechler

**Affiliations:** 1 Department of Internal Medicine I, Gastroenterology, Hepatology, Endocrinology, Rheumatology, and Infectious Diseases, University Hospital Regensburg, 93053 Regensburg, Germany; vlad.pavel@klinik.uni-regensburg.de (V.P.); patricia.mester@klinik.uni-regensburg.de (P.M.); stephan.schmid@klinik.uni-regensburg.de (S.S.); martina.mueller-schilling@klinik.uni-regensburg.de (M.M.); 2 Institute of Clinical Chemistry and Laboratory Medicine, University Hospital Regensburg, 93053 Regensburg, Germany; marcus.hoering@klinik.uni-regensburg.de (M.H.); gerhard.liebisch@klinik.uni-regensburg.de (G.L.)

**Keywords:** COVID-19, lysophosphatidylcholine, sepsis, mortality

## Abstract

**Background/Objectives:** Low levels of lysophosphatidylcholine (LPC) in the blood can be used as a diagnostic marker for sepsis. SARS-CoV-2 infection, a more recent cause of sepsis, shares similarities with non-SARS-CoV-2 sepsis but also exhibits distinct features. We have recently shown that plasma cholesteryl ester levels are higher in patients with SARS-CoV-2 infection than in patients without, and this study analysed whether this may extend to differences in LPC, a bioactive constituent of lipoproteins. **Methods:** The plasma levels of 13 LPC species were measured by flow injection analysis tandem mass spectrometry (FIA-MS/MS) in 157 patients with systemic inflammatory response syndrome (SIRS), sepsis or septic shock. Of these patients, 24 had SARS-CoV-2 infection. **Results:** Patients with SIRS exhibited higher plasma levels of the minor LPC species LPC 15:0 and 22:4 compared to those with sepsis or septic shock. Five LPC species were also reduced in the plasma of 31 patients with liver cirrhosis; therefore, patients with cirrhosis or SIRS were excluded from subsequent analyses. Compared to 76 non-COVID-19 patients with sepsis or septic shock, SARS-CoV-2 infection in 21 patients was associated with significantly higher plasma levels of ten individual LPC species and total LPC concentration. In patients with sepsis/septic shock, LPC species showed negative correlations with procalcitonin and interleukin-6, and positive correlations with gamma-glutamyltransferase and cholesteryl ester levels. In contrast, no significant associations were observed between LPC levels and C-reactive protein, aminotransferases, or free cholesterol. **Conclusions:** Differential LPC levels, despite comparable disease severity, may serve as metabolic biomarkers to distinguish SARS-CoV-2 sepsis from other causes of sepsis and inform targeted therapeutic approaches.

## 1. Introduction

Sepsis is a severe inflammatory condition that can result in significant morbidity and mortality [1,2,3,4,5]. It arises from an uncontrolled immune response to viral, bacterial and fungal infection with an unknown pathogen and site of infection in about 10% of critically ill patients [2,5,6,7].

Lipid metabolism and the activation of lipid signalling pathways contribute to the challenges of sepsis. The role of lipid mediators in the inflammatory and anti-inflammatory responses during sepsis is a crucial factor in the progression of the disease [8,9,10].

Cholesterol, which is carried in lipoproteins such as low-density lipoprotein (LDL) and high-density lipoprotein (HDL) is often reduced in the circulation of critically ill patients [8,10,11,12]. When evaluating lipids as biomarkers, underlying liver cirrhosis must be considered a potential confounder. Patients with cirrhosis typically show reduced levels of lipoproteins [13], leading to lower serum cholesterol concentrations. Moreover, they are at increased risk for infections [14] and sepsis [15], although it remains unclear whether this predisposition translates into higher mortality [8,15,16,17].

Lipoproteins also carry lysophosphatidylcholines (LPCs) [18], which differ in acyl chain length and degree of unsaturation [18,19]. LPC 16:0 is the most abundant species in human plasma, reaching concentrations of up to 200 µM. It is followed by LPC 18:0, LPC 18:1, and LPC 18:2, which are also commonly present. In contrast, other LPC species occur at much lower concentrations, typically ≤ 10 µM [20].

LPC increases the chemotaxis of innate immune cells and bacterial clearance. LPCs can induce the production of inflammatory chemokines and cytokines and were also found to reduce tumour necrosis factor and interleukin (IL)-1 beta production [21]. These divergent findings may be attributable to the specific biological activities of individual LPC species, whereby saturated LPCs promote inflammation, whereas polyunsaturated LPCs exert anti-inflammatory effects [22].

LPC in serum is converted to lysophosphatidic acid by autotaxin [23], and higher circulating autotaxin levels predict mortality of patients with acute respiratory distress syndrome [24] and of patients with severe sepsis [25]. Consistent with increased autotaxin levels, the circulating levels of LPC are low in sepsis. This was shown for total LPC levels and the LPC species 15:0, 16:0, 18:0, 18:1, 18:2, 18:3, 20:3, 20:4, and 20:5 [26,27]. Sepsis patients consistently had reduced levels of LPC compared to healthy donors [28]. LPC levels were also found to discriminate between patients with sepsis and patients with systemic inflammatory response syndrome (SIRS), a less severe state of systemic inflammation [28,29]. Further analysis indicated a more pronounced decrease in total LPC levels in sepsis caused by bacteria in comparison to non-bacterial sepsis. In this cohort, serum LPC was not associated with disease severity or survival [30]. In rodent sepsis models, LPC protected from organ damage and improved outcome [31,32], showing that low LPC may have a role in disease progression.

SARS-CoV-2 infection can also cause sepsis and septic shock [33], which was associated with higher levels of plasma LPC 16:1 compared to healthy donors [34]. LPC 16:0 was found to be reduced in COVID-19 compared to healthy blood donors. Interestingly, patients with COVID-19 sepsis had higher plasma LPC 16:0 levels than patients with sepsis of other causes [35].

In our recent study, patients with SARS-CoV-2 infection exhibited higher plasma levels of cholesteryl esters compared to severely ill patients without viral infection [36], suggesting distinct inflammation-related effects on systemic lipid metabolism.

This study assessed plasma levels of individual LPC species in patients with sepsis or septic shock, with and without SARS-CoV-2 infection. The aim was to evaluate whether sepsis-related metabolic alterations—closely linked to the immune response [6]—differ between these groups, potentially reflecting distinct underlying pathophysiological mechanisms.

## 2. Materials and Methods

### 2.1. Study Cohort

Plasma samples were collected from patients admitted to the medical intensive care unit of the University Hospital Regensburg with a focus on liver, gastrointestinal, and infectious diseases between August 2018 and January 2024. The Sepsis-3 criteria [37] and the systemic inflammatory response syndrome (SIRS) criteria [38] were used to classify the patients for sepsis, septic shock, and SIRS. Plasma samples of COVID-19 patients were gathered from October 2020 to January 2023.

Patients with multidrug-resistant infections, hepatitis virus infections, or human immunodeficiency virus (HIV) infection were excluded from the study. Otherwise, this retrospective analysis included all patients admitted to the intensive care unit who met the inclusion criteria and provided informed consent.

Our patients had different causes of SIRS, sepsis, or septic shock. Common underlying diseases of our patients were liver cirrhosis (20.0%), pancreatitis (20%), neoplasms such as colorectal cancer and adenocarcinoma (13.5%), autoimmune diseases such as Sjögren’s syndrome (7.7%), haematological diseases including acute promyelocytic leukaemia and acute lymphoblastic leukaemia (7.7%). Moreover, 7.0% of our patients were immunosuppressed after organ transplantation. The patients had different underlying diseases, and it was not possible to analyse most of these subgroups separately because they were too small for statistical analysis.

Between 12 and 24 h after being admitted to the intensive care unit, blood samples were taken from the patients. At the same time, blood samples were taken for analysis of LPC species, cholesterol levels and laboratory parameters. Laboratory parameters were obtained from the Institute of Clinical Chemistry and Laboratory Medicine at the University Hospital of Regensburg.

Patients who died during their intensive care unit stay were classified as non-survivors, while those discharged alive were classified as survivors. All patients who did not survive had septic multiorgan failure.

A total of 23 healthy donors (10 males and 13 females), with a median age of 42 years (range 25–78), were recruited from among the clinic’s employees, students, and relatives of students and employees involved in the project. These donors had normal body weight and were all healthy; laboratory parameters were not obtained.

### 2.2. Measurement of Plasma LPC Species

LPC species levels were determined by flow injection analysis electrospray ionization tandem mass spectrometry (FIA-ESI-MS/MS) [39]. A volume of 25 µL of internal standard solution containing LPC 13:0 (0.83 nmol) and LPC 19:0 (0.7 nmol) was added prior to lipid extraction. A sample volume of 10 µL plasma was extracted according to the protocol of Bligh and Dyer [40]. The chloroform phase was vacuum-dried, solubilized in 7.5 mM ammonium acetate in methanol-chloroform (3:1 by volume). Using a product ion of *m*/*z* 184, the triple quadrupole mass spectrometer (Quattro LC; Micromass; Manchester, UK) was run in positive selected reaction monitoring. Matrix-based calibration lines were created using 1-acyl-2-hydroxy-sn-glycero-3-phosphocholines, LPC 16:0, 18:0, and 18:1 (Avanti Polar Lipids, Alabaster, AL, USA). Methods used for the quantification of plasma cholesterol have been described before [36,41].

### 2.3. Statistical Analysis

The median, first and third quartiles, and minimum and maximum levels are mapped out in box plots, which are used to display the data. Asterisks or single circles represent outliers. The median, minimum, and maximum values of each dataset are displayed in tabular form. Statistical analyses were performed using IBM SPSS Statistics 26.0 (IBM Corp., Armonk, NY, USA; released 2019). LPC species levels in the plasma of our patients did not follow a normal distribution (Shapiro–Wilk test: *p* < 0.001 for all). To assess relationships between continuous variables, the following tests were employed: (1) the non-parametric Mann–Whitney U test for two-group comparisons; (2) the non-parametric Kruskal–Wallis test with post hoc Bonferroni correction for multiple-group comparisons; (3) the chi-squared test for categorical variables; (4) Spearman’s correlation to evaluate relationships between continuous variables; (5) receiver-operating characteristic curve. A *p*-value < 0.05 was considered significant. The *p*-values were not corrected for multiple comparisons. Whether or not adjustment for multiple comparisons is advisable remains a matter of debate [42].

## 3. Results

### 3.1. LPC Species Levels of Patients with and Without Liver Cirrhosis and Comparison with Healthy Donors

Liver cirrhosis was relatively common in our cohort, and 31 of the 157 patients with SIRS/sepsis/septic shock had liver cirrhosis. LPC 16:0, 20:3, 20:4, 20:5, and 22:6 were reduced in the plasma of patients with cirrhosis in comparison with non-cirrhotic patients (Table S1). Hence, patients with liver cirrhosis were excluded from further analyses.

The 126 patients with SIRS/sepsis/septic shock without liver cirrhosis had greatly reduced levels of all LPC species in comparison to the 23 healthy donors (10 males and 13 females) (Table 1). The median age of the healthy donors was 42 (25–78) years, and they were younger than the patients (*p* < 0.001).

Pancreatitis was also a common underlying disease. However, patients with and without pancreatitis had similar levels of all LPC species in comparison to patients without pancreatitis (*p* > 0.05 for all).

### 3.2. LPC Species Levels of Patients with and Without SARS-CoV-2 Infection and Stratification for SIRS—Sepsis—Septic Shock

**Figure 1:**
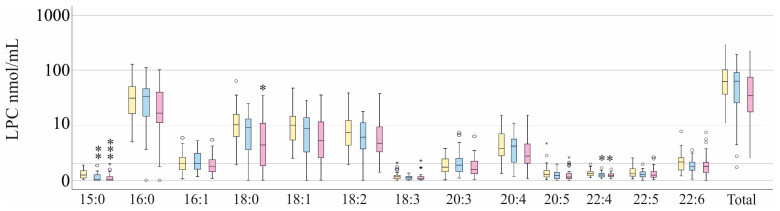
LPC species in plasma of patients with systemic inflammatory response syndrome (SIRS), sepsis, or septic shock. Patients with liver cirrhosis and patients with COVID-19 were excluded. LPC species in plasma of patients with SIRS (yellow bars), sepsis (blue bars), or septic shock (pink bars). Outliers are represented by circles and small asterisks. Kruskal–Wallis test: * *p* < 0.05, ** *p* < 0.01, *** *p* < 0.001 in comparison to patients with SIRS.

Next, we calculated whether LPC species levels differ between patients with SIRS, sepsis, and septic shock. For this analysis, patients with liver cirrhosis and patients with COVID-19 were excluded. LPC 15:0 and LPC 22:4 were higher in patients with SIRS compared to those with sepsis and to those with septic shock. LPC 18:0 was increased in SIRS compared to septic shock (Figure 1). The areas under the receiver-operating characteristic curve were 0.213 ± 0.052 (*p* < 0.001), 0.293 ± 0.060 (*p* = 0.003) and 0.348 ± 0.065 (*p* = 0.029) for LPC 15:0, 18:0 and 22:4, respectively, to discriminate SIRS and septic shock.

As none of the patients with SARS-CoV-2 infection had SIRS, patients with SIRS were excluded from comparisons between patients with and without SARS-CoV-2 infection. Table 2 describes the details of the SIRS/sepsis/septic shock patients (excluding those with SARS-CoV-2 infection), the sepsis/septic shock patients without SARS-CoV-2 infection, and the patients with SARS-CoV-2 infection (who all had either sepsis or septic shock).

Sepsis/septic shock patients with COVID-19 had lower C-reactive protein (CRP), procalcitonin, IL-6, basophils, and eosinophils compared to sepsis/septic shock patients of other causes. Albumin and aminotransferase levels of the COVID-19 patients were higher. Total bilirubin and gamma-glutamyltransferase were similar between the groups (Table 2). Sex distribution, age, body mass index, leukocytes, neutrophils, monocytes, lymphocytes and immature granulocytes did not differ between the groups (Table 2).

**Figure 2:**
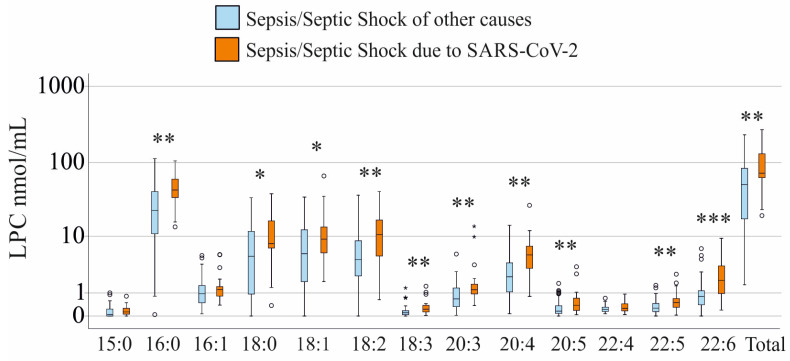
LPC species in plasma of patients with sepsis or septic shock without (blue bars) and with SARS-CoV-2 infection (orange bars). Patients with liver cirrhosis were excluded. Circles and small asterisks represent outliers. Mann–Whitney U test: * *p* < 0.05, ** *p* < 0.01, *** *p* < 0.001.

COVID-19 patients had higher plasma LPC 16:0, 18:0, 18:1, 18:2, 18:3, 20:3, 20:4, 20:5, 22:5, 22:6, and total LPC levels compared to sepsis patients of other causes (Figure 2).

In comparison to the healthy donors, levels of all LPC species and total LPC were lower in patients with COVID-19 (*p* < 0.001 for all).

### 3.3. Correlation of LPC Species Levels with Markers of Inflammation

Combining patients with underlying diseases that are known to affect LPC levels is not advisable for correlation analysis, as this may lead to incorrect results [43]. Therefore, for correlation analysis, patients with liver cirrhosis (who had low levels of several LPC species) and patients with COVID-19 (who had higher levels of most LPC species) were excluded for this analysis.

It should be noted that LPC species did not correlate with age and did not differ between sexes (*p* > 0.05 for all), showing that these factors do not need to be considered as confounders.

In patients with SIRS/sepsis/septic shock, LPC 15:0 negatively correlated with CRP, and all but LPC 18:3, 22:4, 22:5, and 22:6 with procalcitonin. IL-6 negatively correlated with all LPC species (Table 3).

As patients with SIRS had higher levels of LPC 15:0, LPC 18:0, and LPC 22:4 than patients with sepsis/septic shock (Figure 1), correlation analysis was also performed on the subgroup of patients with sepsis/septic shock, excluding those with SIRS.

The correlation of LPC 15:0 with CRP was not significant, whereas all other significant correlations remained significant when patients with SIRS were excluded (Table 3).

None of the LPC species correlated with immune cell count in SIRS/sepsis/septic shock and sepsis/septic shock cohorts (*p* > 0.05 for all). In the COVID-19 cohort, correlations of LPC species with CRP, procalcitonin, and IL-6 were not significant (*p* > 0.05 for all).

### 3.4. Correlation of LPC Species Levels with Markers of Liver Disease

For correlation analysis, patients with liver cirrhosis and patients with COVID-19 were excluded. In patients with SIRS/sepsis/septic shock LPC 16:0, 20:4, 20:5, 22:6, and total LPC levels negatively correlated with total bilirubin (Table 4). LPCs did not correlate with aminotransferase or albumin levels. All but LPC 20:4 and 22:4 positively correlated with gamma-glutamyltransferase (Table 4).

In patients with sepsis/septic shock LPC 16:0, 18:0, 20:4, 20:5, 22:4, 22:5 and 22:6 as well as total LPC levels negatively correlated with total bilirubin (Table 4). LPCs did not correlate with aminotransferase or albumin levels (*p* > 0.05 for all). All but LPC 20:5 and 22:4 positively correlated with gamma-glutamyltransferase (Table 4).

Nine of our patients had cholangiosepsis with increased levels of gamma-glutamyltransferase (*p* = 0.001), but LPC species levels of these patients were not induced (*p* > 0.05 for all).

### 3.5. Correlation of LPC Species Levels with Cholesterol

Patients with liver cirrhosis (who had low levels of most LPC species) and patients with COVID-19 (who had higher levels of several LPC species) were excluded from the correlation analysis. In patients with SIRS/sepsis/septic shock and in patients with sepsis/septic shock, LPC species levels positively correlated with cholesteryl ester levels and total plasma cholesterol (Table 5). There was a modest correlation of LPC 18:3 with free cholesterol in the entire cohort (Table 5).

Total cholesterol and cholesteryl ester levels of patients with sepsis/septic shock due to COVID-19 were higher than those of sepsis/septic shock patients without this virus (Table 6).

### 3.6. LPC Species Levels and Survival

**Figure 3:**
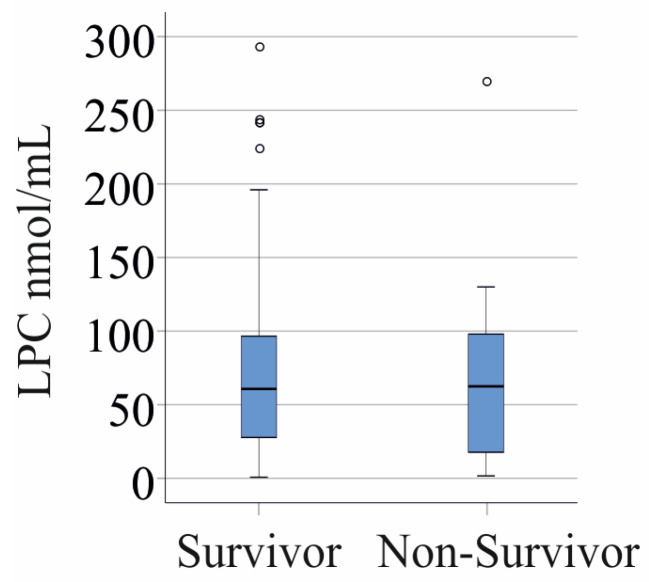
LPC levels in the plasma of SIRS/sepsis/septic shock patients who survived and those who died because of septic multiorgan failure. Outliers are represented by circles. Patients with liver cirrhosis were excluded.

Among the 25 SIRS/sepsis/septic shock patients who did not survive (patients with liver cirrhosis were excluded), total LPC concentrations and individual LPC species levels were comparable to those of survivors (*p* > 0.05 for all) (Figure 3). Similarly, in the non-COVID-19 cohort, LPC levels did not differ significantly between survivors and the 18 non-survivors (patients with liver cirrhosis were excluded).

## 4. Discussion

This study supports previous observations that plasma LPC levels are markedly reduced in patients with sepsis or septic shock compared to healthy individuals [26,44,45]. Notably, patients with SARS-CoV-2 infection showed a less pronounced decline in LPCs despite similar disease severity, suggesting distinct patterns of lipid dysregulation in viral versus non-viral sepsis.

Reduced levels of LPCs are a common finding in patients with inflammation [26,44,45]. LPCs in the blood are bound to albumin and lipoproteins [18]. In addition to LPC, albumin and cholesterol levels in plasma are reduced in severe inflammation [36,46,47]. Current correlation analysis suggests that it is low cholesterol, rather than reduced levels of albumin, that is related to the decline of LPCs.

Reduced cholesterol levels are a known consequence of systemic inflammation and tend to normalise as inflammation resolves [46,48], highlighting the link between inflammation and hypolipidemia. In our study, patients with SARS-CoV-2 infection had lower levels of inflammatory markers such as CRP, procalcitonin, and IL-6 compared to non-COVID-19 patients, suggesting that reduced inflammatory burden may contribute to the higher cholesterol and LPC levels observed in this group.

Cholesteryl ester and LPC levels showed a negative correlation with IL-6 and procalcitonin suggesting a role for these proteins in lipid regulation [36]. While an association between cholesterol and procalcitonin has not been previously reported, IL-6 inhibition in rheumatoid arthritis is known to increase serum cholesterol levels [49]. IL-6 promotes LDL receptor expression and hepatic LDL uptake, contributing to lower plasma LDL and overall cholesterol levels [50]. Thus, lower IL-6 levels in SARS-CoV-2 patients may account for higher plasma cholesterol and LPC concentrations. Consistent with this, all LPC species in our cohort were negatively correlated with IL-6, aligning with previous findings for LPC 16:0 in sepsis [35].

In sepsis, procalcitonin is released by various cells and tissues, including the liver [51], and, like CRP [52], its production is induced by IL-6, alongside other inflammatory cytokines and lipopolysaccharide [51]. The reason why LPCs correlate with procalcitonin but not with CRP is unclear.

All of the cases with confirmed SARS-CoV-2 infection had sepsis or septic shock, and several laboratory measures differed when compared to patients with sepsis/septic shock caused by other factors. Patients with SARS-CoV-2 infection had lower CRP, procalcitonin and IL-6 levels in accordance with previous studies [53,54,55,56]. Patients with SARS-CoV-2 infection had reduced basophil and eosinophil numbers, and a reduced basophil count of these patients has been reported before. Our patients with SARS-CoV-2 infection had higher AST and ALT levels than non-SARS-CoV-2 sepsis patients, with median values almost within the normal range. Lower AST and normal ALT levels were found in patients with SARS-CoV-2-associated sepsis as well [57]. The different findings regarding measures of liver function may be partly due to underlying diseases, which need further study.

The inflammatory markers procalcitonin and CRP are reduced in patients with liver cirrhosis [51,58], a group excluded from our study. Among patients with SIRS, sepsis, or septic shock five of the thirteen measured LPC species were significantly reduced in cirrhosis in line with previous findings in non-septic patients [59]. It is important to demonstrate that the association between cirrhosis and low LPC levels is also evident in cases of sepsis, as this could improve our understanding of this condition.

LPC species did not correlate with AST, ALT, or albumin levels, indicating that plasma levels are not closely related to liver function in patients without cirrhosis. Notably, LPC species with fatty acids having lower numbers of carbon atoms mostly positively correlated with GGT, an enzyme reflecting biliary diseases [60]. The upper limit for GGT is <40 U/L for females and <60 U/L for males, and was exceeded in most of our patients. Patients with cholecystolithiasis have higher levels of LPC 16:0 and 20:1 in their serum than healthy donors, indicating that serum LPC levels may be induced in biliary diseases [61]. However, most LPC species in patients with SIRS, sepsis, or septic shock did not or had a negative correlation with bilirubin, which is also induced in biliary disease [62]. Moreover, patients with cholangiosepsis did not have higher levels of plasma LPCs. This shows that the positive correlation between LPC species and GGT does not result in higher LPC plasma levels in patients with cholestasis, and that the underlying association is unclear.

In our cohort, the levels of cholesteryl esters in patients with SARS-CoV-2 infection were higher than in patients without [36]. LPC species were positively correlated with cholesterol, and suggests that higher levels of LPC are a result of higher cholesterol in patients with COVID-19. Trovato et al. demonstrated that patients with COVID-19 sepsis had significantly higher levels of LPC 16:0 than patients with sepsis due to other causes, in accordance with our findings [35].

An interesting finding of our study was that LPC 15:0, 18:0, and 22:4 were higher in patients with SIRS than in those with sepsis/septic shock, showing that these LPC species decline with disease severity. However, the differences were too small to recommend these lipid species as diagnostic tools.

This study has limitations. The progression of the disease prior to admission to the intensive care unit was not documented. Plasma was collected upon admission to the intensive care unit, and we cannot provide any information on LPC plasma levels throughout the course of the disease. For most patients, antibiotic therapy had begun before blood samples were taken, which may have affected systemic LPC levels. LPC is produced by phospholipase A2 [22,63], whose levels increase in sepsis [64] and decrease following steroid therapy [65]. Phospholipase A2 may also emerge as a drug target for the treatment of sepsis in patients with SARS-CoV-2 infection [66]. Because LPC levels are low in sepsis, the role of phospholipase A2 and drugs that reduce phospholipase A2 for systemic LPC levels requires further study. However, the current study has not analysed the associations of LPC levels with medications such as steroids.

Further research is recommended to investigate the relationship between different drugs and systemic levels of LPC. Proposed longitudinal studies would evaluate whether plasma LPC levels normalise during patient recovery, which may make them a potential biomarker for outcome. Although LPC levels decrease in sepsis, levels analysed early in the disease course are not useful for prediction of the outcome.

## 5. Conclusions

This analysis demonstrated that most LPC species were elevated in the plasma of sepsis/septic shock patients with SARS-CoV-2 infection compared to those with non-COVID-19 sepsis. These findings were consistent with lower levels of CRP, procalcitonin, and IL-6, and negative correlations between LPCs and the latter two inflammatory markers. Our results support a distinct inflammatory and metabolic profile in SARS-CoV-2 sepsis and reinforce the inverse relationship between systemic inflammation and circulating lipid levels.

## Figures and Tables

**Table 1:** Median, minimum, and maximum LPC levels, and 95% confidence interval (CI) of patients, excluding those with liver cirrhosis, and healthy donors.

LPC nmol/mL	Healthy Donors n = 23		Patients n = 126		*p*-Value
	Median (Minimum- Maximum	95% CI	Median (Minimum- Maximum	95% CI	
15:0	1.68 (1.06–3.98)	1.53–2.12	0.11 (0.00–0.97)	0.15–0.23	<0.001
16:0	139.33 (82.36–248.97)	129.72–166.24	32.18 (0.00–128.58)	30.46–40.59	<0.001
16:1	4.28 (2.16–11.22)	3.71–5.33	1.01 (0.07–4.93)	1.11–1.48	<0.001
18:0	46.41 (19.37–105.06)	43.12–61.18	7.38 (0.00–65.34)	7.80–11.29	<0.001
18:1	40.86 (15.16–61.57)	34.36–45.12	7.58 (0.00–65.11)	8.28–11.95	<0.001
18:2	54.93 (17.90–84.73)	51.07–66.31	5.95 (0.00–41.10)	7.22–10.47	<0.001
18:3	1.11 (0.37–3.46)	1.04–1.62	0.13 (0.00–1.41)	0.16–0.24	<0.001
20:3	4.76 (1.89–8.99)	4.21–5.49	0.80 (0.02–12.82)	0.97–1.54	<0.001
20:4	11.18 (6.36–20.23)	10.21–13.75	2.78 (0.07–26.34)	3.37–4.81	<0.001
20:5	1.16 (0.47–5.92)	0.98–1.94	0.24 (0.00–3.69)	0.31–0.50	<0.001
22:4	0.72 (0.31–1.39)	0.61–0.90	0.26 (0.04–0.96)	0.27–0.33	<0.001
22:5	1.03 (0.34–1.70)	0.88–2.00	0.32 (0.00–2.34)	0.36–0.49	<0.001
22:6	2.56 (1.31–5.93)	2.25–3.23	0.96 (0.00–9.32)	1.13–1.67	<0.001
Total LPC	310.92 (154.67–500.44)	290.48–367.65	62.05 (0.72–293.05)	62.57–84.61	<0.001

**Table 2:** Characteristics of systemic inflammatory response syndrome (SIRS)/sepsis/septic shock patients excluding patients with liver cirrhosis. The details of patients with SIRS/sepsis/septic shock, of patients with sepsis/septic shock (both cohorts excluding patients with COVID-19), and of COVID-19 patients (who did not have SIRS) are listed. Numbers in superscript refer to patients for whom these data were available when data were not collected from the entire cohort. Data are given as median (minimum–maximum) and 95% confidence interval (CI). Statistical tests used: Mann–Whitney U test and chi-squared test. * and ^&^ *p* < 0.05, ^&&^ *p* < 0.01, *** *p* < 0.001.

Parameters	SIRS/Sepsis/Septic Shock Patients Without SARS-CoV-2	Sepsis/Septic Shock Patients Without SARS-CoV-2	Sepsis/Septic Shock Patients with SARS-CoV-2
Males/Females	73/32 (n = 105)	53/23 (n = 76)	15/6 (n = 21)
Age, years	59 (21–93)	59 (21–93)	63 (29–80)
	95% CI: 55–61	95% CI: 54–61	95% CI: 52–65
Body mass index, kg/m^2^	26.2 (15.4–55.6) ^103^	26.5 (15.4–55.6) ^76^	28.3 (23.4–45.3)
	95% CI: 26.9–30.09	95% CI: 27.6–31.6	95% CI: 27.4–33.6
SIRS/Sepsis/Septic shock	29/31/45 ***	0/31/45 ^&&^	0/2/19 *** ^&&^
C-reactive protein, mg/L	187 (23–697)	188 (39–697) ^&^	156 (44–472) ^&^
	95% CI: 188–237	95% CI: 192–252	95% CI: 118–211
Procalcitonin, ng/mL	2.06 (0.05–270.00) ^101^	2.2 (0.06–114.40) ^73 &^	0.57 (0.08–65.40) * ^&^
	95% CI: 7.65–22.27	95% CI: 5.14–15.35	95% CI: −2.24–10.63
Interleukin-6, pg/mL	97 (0–5702) ^102^ *	99 (7–5702) ^74 &^	47 (6–1810) ^20^ * ^&^
	95% CI: 399–850	95% CI: 333–820	95% CI: −0.15–443.93
Leukocytes, n/nL	10.40 (0.06–246.94)	10.40 (0.28–246.94)	9.62 (2.78–18.47)
	95% CI: 9.46–19.15	95% CI: 8.94–21.67	95% CI: 8.94–21.67
Neutrophils, n/nL	7.87 (0–70.20) ^100^	8.86 (0–70.20) ^74^	6.36 (0–48.40) ^146^
	95% CI: 8.88–13.05	95% CI: 9.01–14.35	95% CI: 3.98–12.95
Basophils, n/nL	0.04 (0–0.90) ^101^ *	0.04 (0–0.90) ^75 &^	0.02 (0–0.60) * ^&^
	95% CI: 0.05–0.10	95% CI: 0.06–0.12	95% CI: 0.0–0.12
Eosinophils, n/nL	0.15 (0–1.75) ^101^ *	0.16 (0–1.75) ^75 &^	0.05 (0–8.80) * ^&^
	95% CI: 0.21–0.34	95% CI: 0.20–0.36	95% CI: −0.36–1.37
Monocytes, n/nL	0.79 (0–45.00) ^101^	0.80 (0–45.00) ^75^	0.52 (0–10.90)
	95% CI: 0.54–2.36	95% CI: 0.49–2.92	95% CI: 0.10–2.21
Lymphocytes, n/nL	1.09 (0.08–16.80) ^101^	1.20 (0.08–16.80) ^75^	0.69 (0.08–28.60)
	95% CI: 1.03–1.73	95% CI: 1.04–1.97	95% CI: −0.17–5.36
Immature granulocytes, n/nL	0.10 (0–6.19) ^99^	0.20 (0–6.19) ^74^	0.19 (0–3.84)
	95% CI: 0.31–0.75	95% CI: 0.39–0.97	95% CI: 0.09–0.96
Total bilirubin, mg/dL	0.80 (0.10–23.90) ^99^ *	0.80 (0.20–23.90) ^72^	0.60 (0.20–2.80) ^20^ *
	95% CI: 1.50–3.15	95% CI: 1.44–3.61	95% CI: 0.51–1.56
Albumin, g/L	22.1 (6.3–42.0) ^97^ ***	23.1 (6.3–42.0) ^72 &&^	28.1 (20.2–36.6) ^19^ *** ^&&^
	95% CI: 20.9–23.6	95% CI: 20.9–24.1	95% CI: 23.5–29.9
AST, U/L	39 (6–1597) ^93^	38 (8–1597) ^66 &^	58 (23–126) ^20 &^
	95% CI: 43–156	95% CI: 29–148	95% CI: 41–86
ALT, U/L	30 (6–770) ^94^	27 (6–770) ^67 &^	42 (13–283) ^17 &^
	95% CI: 35–84	95% CI: 23–87	95% CI: 20–116
GGT, U/L	117 (11–1093) ^87^	114 (11–704) ^63^	229 (22–1266) ^13^
	95% CI: 147–239	95% CI: 107–180	95% CI: 45–469

Alanine aminotransferase: ALT; Aspartate aminotransferase; AST; Gamma-glutamyltransferase: GGT.

**Table 3:** Spearman’s correlation coefficients for the correlation of LPC species and total LPC levels with C-reactive protein (CRP), procalcitonin, and interleukin-6 (IL-6) in patients with SIRS/sepsis/septic shock and in the subgroup of patients with sepsis/septic shock. Patients with liver cirrhosis and patients with COVID-19 were excluded. * *p* < 0.05, ** *p* < 0.01, *** *p* < 0.001.

LPC	CRP	Procalcitonin	IL-6	CRP	Procalcitonin	IL-6
	SIRS/Sepsis/Septic Shock			Sepsis/Septic Shock		
15:0	−0.211 *	−0.357 **	−0.338 **	−0.169	−0.441 ***	−0.237 *
16:0	0.000	−0.509 ***	−0.531 ***	0.084	−0.516 ***	−0.451 ***
16:1	−0.143	−0.385 **	−0.465 ***	−0.072	−0.401 ***	−0.392 **
18:0	0.006	−0.443 ***	−0.375 **	0.100	−0.437 ***	−0.260 *
18:1	−0.115	−0.414 ***	−0.443 ***	−0.051	−0.413 ***	−0.354 **
18:2	−0.112	−0.360 **	−0.467 ***	−0.027	−0.344 **	−0.370 **
18:3	−0.132	−0.209	−0.376 **	−0.022	−0.203	−0.250 *
20:3	−0.073	−0.399 ***	−0.450 ***	0.030	−0.396 **	−0.355 **
20:4	−0.078	−0.421 ***	−0.526 ***	0.007	−0.412 ***	−0.479 ***
20:5	−0.076	−0.291 *	−0.408 ***	0.042	−0.309 **	−0.308 **
22:4	−0.111	−0.102	−0.394 **	−0.037	−0.087	−0.349 **
22:5	0.048	−0.180	−0.400 ***	0.175	−0.158	−0.337 **
22:6	−0.031	−0.208	−0.463 ***	0.097	−0.181	−0.375 **
Total LPC	−0.034	−0.476 ***	−0.500 ***	0.058	−0.483 ***	−0.407 ***

**Table 4:** Spearman’s correlation coefficients for the correlation of LPC species with total bilirubin, aspartate aminotransferase (AST), alanine aminotransferase (ALT), gamma glutamyltransferase (GGT) and albumin in SIRS/sepsis/septic shock patients and in the subgroup of patients with sepsis/septic shock. Patients with liver cirrhosis and patients with COVID-19 were excluded. * *p* < 0.05, ** *p* < 0.01, *** *p* < 0.001.

LPC	Bilirubin Total	AST	ALT	GGT	Albumin	Bilirubin Total	GGT
	SIRS/Sepsis/Septic Shock					Sepsis/Septic Shock	
15:0	−0.187	−0.117	−0.020	0.363 **	−0.044	−0.163	0.302 *
16:0	−0.392 **	−0.199	−0.138	0.334 **	0.109	−0.417 ***	0.325 **
16:1	−0.214	−0.065	0.020	0.488 ***	−0.019	−0.228	0.461 ***
18:0	−0.265	−0.155	−0.049	0.400 **	−0.030	−0.244 *	0.393 **
18:1	−0.224	−0.101	0.003	0.479 ***	−0.053	−0.200	0.449 ***
18:2	−0.185	−0.015	0.093	0.488 ***	0.019	−0.112	0.471 ***
18:3	−0.088	0.061	0.129	0.548 ***	0.050	−0.034	0.480 ***
20:3	−0.283	−0.089	0.024	0.478 ***	0.032	−0.224	0.504 ***
20:4	−0.397 **	−0.122	0.005	0.297	0.093	−0.377 **	0.280 *
20:5	−0.394 **	−0.096	−0.020	0.333 *	0.109	−0.418 ***	0.229
22:4	−0.279	−0.060	−0.001	0.099	0.010	−0.331 **	−0.064
22:5	−0.276	−0.025	0.050	0.320 *	0.096	−0.271 *	0.251 *
22:6	−0.320 *	−0.028	0.050	0.338 *	0.111	−0.295 *	0.263 *
Total LPC	−0.341 **	−0.155	−0.072	0.407 **	0.037	−0.342 **	0.392 **

**Table 5:** Spearman’s correlation coefficients for the correlation of LPC species with cholesteryl ester levels, free cholesterol levels, and total cholesterol in the SIRS/sepsis/septic shock patients and in the subgroup of patients with sepsis/septic shock. Patients with liver cirrhosis and patients with COVID-19 were excluded. * *p* < 0.05, ** *p* < 0.01, *** *p* < 0.001.

LPC	Cholesteryl Ester	Free Cholesterol	Total Cholesterol	Cholesteryl Ester	Free Cholesterol	Total Cholesterol
	SIRS/Sepsis/Septic Shock			Sepsis/Septic Shock		
15:0	0.617 ***	0.035	0.513 ***	0.551 ***	0.059	0.461 ***
16:0	0.741 ***	0.157	0.645 ***	0.729 ***	0.166	0.615 ***
16:1	0.526 ***	0.162	0.486 ***	0.529 ***	0.124	0.445 ***
18:0	0.685 ***	0.145	0.595 ***	0.634 ***	0.178	0.548 ***
18:1	0.565 ***	0.164	0.508 ***	0.528 ***	0.171	0.465 ***
18:2	0.504 ***	0.175	0.470 ***	0.478 ***	0.225	0.460 ***
18:3	0.357 ***	0.198 *	0.375 ***	0.285 *	0.223	0.320 **
20:3	0.560 ***	0.091	0.488 ***	0.544 ***	0.116	0.484 ***
20:4	0.657 ***	0.057	0.523 ***	0.675 ***	0.082	0.528 ***
20:5	0.549 ***	0.087	0.453 ***	0.543 ***	0.066	0.405 ***
22:4	0.438 ***	−0.071	0.290 **	0.453 ***	−0.130	0.243 *
22:5	0.517 ***	0.153	0.472 ***	0.484 ***	0.131	0.420 ***
22:6	0.525 ***	0.144	0.459 ***	0.523 ***	0.158	0.445 ***
Total LPC	0.681 ***	0.156	0.594 ***	0.662 ***	0.172	0.564 ***

**Table 6:** Median, minimum, and maximum cholesterol levels and 95% confidence interval (CI) of sepsis/septic shock patients with and without COVID-19.

Cholesterol nmol/mL	Without SARS-CoV-2 (n = 76)		With SARS-CoV-2 (n = 21)		*p*-Value
	Median (Minimum- Maximum	95% CI	Median (Minimum- Maximum	95% CI	
Free cholesterol	1142 (424–5098)	1177–1539	1154 (505–2642)	1001–1450	>0.05
Cholesteryl ester	1136 (138–3558)	1082–1471	1853 (709–3668)	1534–2231	0.002
Total cholesterol	2228 (1097–8529)	2333–2936	2976 (1290–5727)	2603–3613	0.026

## Data Availability

Data are shown in the manuscript.

## Supplementary Materials

The following supporting information can be downloaded at: https://www.mdpi.com/article/10.3390/biomedicines13092110/s1, Table S1: Median, minimum, and maximum LPC levels and 95% confidence interval (CI) of patients with and without liver cirrhosis.

## Abbreviations

The following abbreviations are used in this manuscript:

ALT	Alanine aminotransferase
AST	Aspartate aminotransferase
CRP	C-reactive protein
GGT	Gamma-glutamyltransferase
Interleukin	IL
LPC	Lysophosphatidylcholine
SARS-CoV-2	Severe acute respiratory syndrome coronavirus 2
SIRS	Systemic inflammatory response syndrome
