# Supplementary material for: Distinct Plasma LPC Signatures Differentiate COVID-19 Sepsis from Other Sepsis Aetiologies
Source: Biomedicines. 2025 Aug 29;13(9):2110. doi: 10.3390/biomedicines13092110 (PMC12466981; doi:10.3390/biomedicines13092110)
Supplement: Supplementary file 1 [file biomedicines-13-02110-s001.zip › biomedicines-3830538-supplementary.pdf]

Table S1. Median, minimum and maximum LPC levels and 95% confidence interval (CI) of patients with and without liver cirrhosis

LPC nmol/ml	Without Liver Cirrhosis n = 126		With Liver Cirrhosis n = 31		P-value
	Median (Minimum-Maximum	95% CI	Median (Minimum-Maximum	95% CI	
15:0	0.11 (0.00–0.97)	0.15–0.23	0.12 (0.00–0.55)	0.11–0.20	> 0.05
16:0	32.18 (0.00–128.58)	30.46–40.59	13.65 (3.38–58.85)	11.80–21.50	0.003
16:1	1.01 (0.07–4.939)	1.11–1.48	0.90 (0.20–3.24)	0.77–1.31	> 0.05
18:0	7.38 (0.00–65.34)	7.80–11.29	4.51 (0.00–21.49)	3.79–7.05	> 0.05
18:1	7.58 (0.00–65.11)	8.28–11.96	6.81(1.81–21.50)	6.33–10.34	> 0.05
18:2	5.95 (0.00–41.10)	7.22–10.47	4.76 (0.99–20.67)	4.22–7.85	> 0.05
18:3	0.13 (0.00–1.41)	0.16–0.24	0.09 (0.02–0.69)	0.08–0.18	> 0.05
20:3	0.80 (0.02–12.82)	0.97–1.54	0.40 (0.13–2.79)	0.36–0.73	0.003
20:4	2.78 (0.07–26.34)	3.37–4.81	1.60 (0.40–4.87)	1.38–2.22	0.012
20:5	0.24 (0.00–3.69)	0.31–0.50	0.11 (0.01–0.84)	0.09–0.20	0.003
22:4	0.26 (0.04–0.96)	0.27–0.33	0.29 (0.11–0.55)	0.27–0.34	> 0.05
22:5	0.32 (0.00–2.34)	0.36–0.49	0.21 (0.07–0.48)	0.19–0.27	> 0.05
22:6	0.96 (0.00–9.32)	1.13–1.67	0.49 (0.13–2.04)	0.43–0.73	0.002
Total LPC	62.05 (0.72–293.05)	62.57–84.61	34.58 (9.87–130.83)	30.93–51.82	> 0.05
